# Dialectical Versus Linear Thinking Shapes People’s Anticipation of Climate Change

**DOI:** 10.3389/fpsyg.2020.623591

**Published:** 2021-01-20

**Authors:** Liman Man Wai Li, Dongmei Mei, Wen-Qiao Li, Kenichi Ito

**Affiliations:** ^1^Department of Psychology, Centre for Psychosocial Health, The Education University of Hong Kong, Tai Po, Hong Kong; ^2^School of Psychology, Guizhou Normal University, Guiyang, China; ^3^Department of Behavioral Science, Hokkaido University, Sapporo, Japan; ^4^School of Social Sciences, Nanyang Technological University, Singapore, Singapore

**Keywords:** dialectical beliefs, climate change, cross-cultural study, culture, perception of change

## Abstract

Dialectical thinking refers to a constellation of beliefs that consist of expectation of change, tolerance of contradiction, and holism. The current research explored whether dialectical thinking would affect people’s anticipation of climate change, which has been propagated globally. Study 1 compared the responses between Chinese participants, representing people from cultures that promote dialectical thinking, and North American participants, representing people from cultures that promote linear thinking. The results showed that Chinese participants demonstrated a stronger non-linear pattern regarding the anticipation of climate change as compared with American participants, in which Chinese participants were more likely to anticipate a stable trend but less likely to anticipate an increasing trend for global warming. Study 2 with a manipulation of dialectical and linear thinking was conducted and provided some generally supportive evidence for the causal relation between dialectical beliefs and the anticipation of climate change. Implications for cross-cultural environmental research and international climate change education programs were discussed.

## Introduction

Climate change has been treated as one important topic on the global agenda in the contemporary era (Dunlap et al., 2000). However, people still fail to take immediate actions despite many worldwide international pro-environmental campaigns (Kashima et al., 2014). One reason that causes inaction for this environmental crisis is that people feel uncertain whether climate change occurs and continues (Ecklund et al., 2017).

The perception of climate change can be attributed to some individual characteristics, such as religious ideology (e.g., Smith and Leiserowitz, 2013), political ideology (e.g., Davidson and Haan, 2012), and confidence in science (Ecklund et al., 2017). Individuals’ perception of climate change can be also likely to be affected by socio-ecological contexts, which are found to substantially shape how people think, believe, and behave across different domains (e.g., Markus and Kitayama, 1991; Nisbett and Masuda, 2003; Leung and Bond, 2004) including responses to environmental issues (e.g., Eom et al., 2016). However, similar to other disciplines (Henrich et al., 2010), most environmental studies were conducted mainly in western, educated, industrial, rich, and democratic (WEIRD) societies. In addition, many previous cross-cultural environmental studies compared responses from two or multiple cultures (e.g., Milfont et al., 2010; Eom et al., 2016; Pisano and Lubell, 2017; Tam and Chan, 2017), which can differ in multiple dimensions (e.g., Li et al., 2016). Comparing responses in different cultures may not allow us to single out which factor(s) can attribute to the observed cultural differences (Fischer and Poortinga, 2018). More importantly, causal evidence was often lacking even when researchers have successfully identified some important factors associated with people’s responses to environmental issues across cultures.

Given that the perception of climate change has a significant role in affecting people’s engagement in pro-environmental campaigns (Panno et al., 2015), it is crucial to understand what shapes people’s perception of climate change across cultures (Milfont and Schultz, 2016). To extend previous work, the present research conducted two studies to investigate the influence of dialectical thinking, which reflects people’s understanding of the nature of the universe (Peng and Nisbett, 1999; Spencer-Rodgers et al., 2010), on their anticipation of climate change, i.e., the change in global temperature. Study 1 compared the anticipation of climate change between Chinese and American participants, representing people from cultures that promote dialectical thinking and linear (or non-dialectical) thinking, respectively (for a review, see Spencer-Rodgers et al., 2010). And Study 2 activated either dialectical or linear thinking to carefully examine the causal effect of dialectical thinking on people’s perception of climate change.

## Dialectical Thinking and Perception of Change Across Cultures

Dialectical thinking refers to a constellation of lay beliefs about how people understand the nature of the universe (Peng and Nisbett, 1999; Spencer-Rodgers et al., 2010). Given that dialectical beliefs were grounded by Asian ancient philosophy, such as Buddhism and Daoism (Peng and Nisbett, 1999; Spencer-Rodgers et al., 2010), it is not surprising that abundant empirical evidence reveals that dialectical beliefs are more prevalent in East Asian cultures than in North American cultures (e.g., Ng and Hynie, 2014; Li et al., 2016, 2020). Importantly, the influence of dialectical beliefs on a variety of psychological processes and behaviors is observed in both East Asian and North American cultures (e.g., Hamamura et al., 2008; Li et al., 2014; Li, 2018).

Three major principles for dialectical thinking are summarized in previous review studies (Peng and Nisbett, 1999; Spencer-Rodgers et al., 2010). The first principle is expectation of change, which refers to the belief that the universe is full of constant changes. Previous work found that people with dialectical beliefs or from cultures that promote dialectical thinking are more likely to anticipate a non-linear development trend of a target event as compared with people with linear thinking or from cultures that promote linear thinking (Ji et al., 2001). The second principle is tolerance of contradiction, which refers to perceiving that different elements in the universe alternate in their states between two extreme opposites. In line with this principle, it was found that people with dialectical thinking are more likely to perceive that conflicting statements can be equally plausible (Peng and Nisbett, 1999) and to demonstrate ambivalent attitudes (Hamamura et al., 2008). The third principle is holism, which refers to believing that different elements in the universe are highly interdependent and connected. Supporting this, previous work found that people with dialectical thinking perceive more distal and indirect consequences associated with one single target event (Maddux and Yuki, 2006).

The present study focused on the relationship between dialectical thinking and perception of climate change. The endorsement of dialectical beliefs leads to different perceptions of developmental patterns of various target events (Masuda et al., 2018), including stock price (Ji et al., 2008), performance in the competition (Spina et al., 2010b), and socioeconomic status (Li, 2018). In an early work of Ji et al. (2001), Chinese participants, who lived in a culture that promotes dialectical thinking, and American participants, who lived in a culture that promotes linear thinking, were asked to make predictions for economic development. The results showed that Chinese participants were more likely to anticipate a non-linear trend, i.e., a trend differing from the current trend, relative to American participants. Specifically, Chinese participants were more likely to anticipate the pattern of future economic development to be reverse or stable, whereas American participants were more likely to predict the future economic development to continue going up or down following the current economic situation. Spina et al. (2010b) further examined the expectation of future development across different scenarios and found evidence supporting that people from cultures that promote dialectical thinking were likely to predict regression toward the mean, whereas people from cultures that promote linear thinking were likely to predict changes following the current trend. In one scenario, they asked participants to estimate the number of sunny days in a city with a mean of 180 sunny days per year. Chinese participants predicted a lower number of sunny days when last year was hotter than average (i.e., the number of sunny days was higher than the average) but a higher number of sunny days when last year was less hot than average (i.e., the number of sunny days was lower than the average) relative to Canadian participants.

### Dialectical Beliefs and Climate Change

Consistent evidence demonstrated that dialectical beliefs affect how people perceive the future development of various target events (e.g., Ji et al., 2008; Spina et al., 2010b). Following the previous findings, dialectical beliefs may have an influence on people’s anticipation of climate change. However, the judgment of future climate change may not be identical to that for the hypothetical target events adopted previously, as there are substantial differences between the previously examined target events and climate change. In the previously adopted hypothetical scenarios, people usually do not have prior knowledge; thus the expectation of trends can be vulnerable to the influence of other factors such as external socio-cultural factors. In contrast, climate change has become one of the important topics discussed collectively and globally (Dunlap et al., 2000). Therefore, the threat of climate change, i.e., the average temperature will be likely to keep increasing, should have been propagated globally and acquired by most people. It was unknown the extent to which dialectical beliefs may change people’s anticipation of climate change, which is an issue that people may have a lot of prior knowledge of how it may develop in the future. In addition, despite the fact that evidence is accumulated to indicate that dialectical beliefs promote non-linear expectations of future development, except few studies (e.g., Spina et al., 2010a; Li et al., 2018), little work has been done to obtain direct causal evidence.

While an increasing trend of temperature change has been globally propagated, like what has been observed in other domains (e.g., Spina et al., 2010b), dialectical beliefs may foster non-linear predictions against the increasing trend of climate change that is currently propagated. Following previous work (e.g., Ji et al., 2001), dialectical beliefs may make people more likely to predict a stable trend or a declining trend but less likely to predict an increasing trend for future climate change. Therefore, it could be possible that propagating the accelerated severity of global warming may cause undesirable results: people with dialectical beliefs or those from cultures that promote dialectical thinking have more optimistic anticipation of climate change. Some previous findings provided support that dialectical beliefs may have negative influences on people’s responses to environmental issues, in which a negative relation between dialectical beliefs and pro-environmental behaviors were found among Chinese and North American participants (Li et al., 2020).

## Overview of Current Research

To extend the understanding of the influence of culture on the perception of environmental issues, the current research examined the influence of dialectical beliefs on anticipation of climate change, in which people have acquired prior knowledge globally. To obtain initial evidence, participants from China, representing people from cultures that promote dialectical thinking, and from the United States, representing people from cultures that promote linear thinking, were recruited and asked about their anticipation of climate change in Study 1. To provide causal evidence, following previous work (e.g., Li et al., 2014, 2018), we manipulated dialectical vs. linear thinking and observed how dialectical thinking would affect people’s anticipation of climate change in Study 2.

## Study 1

To test the influence of dialectical beliefs on people’s anticipation of climate change, we first conducted a cross-cultural comparison study by recruiting participants from cultures that promote dialectical and linear thinking, respectively.

### Participants

We recruited 83 Chinese (mean age = 19.43, *SD* = 1.95; 53.0% female) from a university in China, representing a culture that promotes dialectical thinking, and 76 Americans (mean age = 35.60, *SD* = 11.54; 39.5% female) through MTurk in the United States, representing a culture that promotes linear thinking. The difference in the level of dialectical thinking between Chinese and North-Americans have been consistently demonstrated in previous work (e.g., Ng and Hynie, 2014; Li et al., 2016, 2020; for a review, see Spencer-Rodgers et al., 2010). Chinese participants participated for partial course credit while American participants participated for US$ 0.50. Based on the calculation using G*Power (Faul et al., 2009), a total sample size of 108 participants was needed to achieve 80% power for a chi-square test with an expected medium effect size (*df* = 2, *p* = 0.05, *w* = 0.30). The research was approved by the Departmental Research Ethics Committee from a university in China. Consent was obtained from the participants before they participated in the study.

### Materials and Procedure

**Figure 1:**
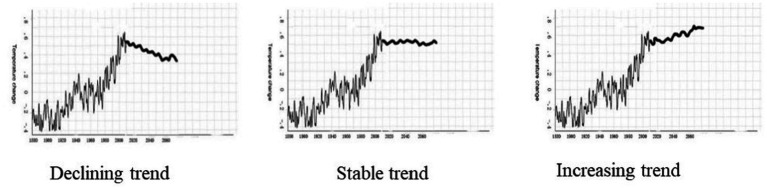
Three options for the anticipation of climate change in Study 1.

The material was presented in Chinese (English) for Chinese (American) participants. Participants were asked to anticipate future climate change (in terms of temperature change) from 2010 to 2060, while the real data from 1880 to 2010, which was in an increasing trend, was presented. They needed to select one of three options that depict a declining trend, a stable trend, or an increasing trend to indicate their anticipation of climate change (see Figure 1). The stable and declining patterns represent non-linear predictions while the increasing pattern represents a linear prediction.

### Results and Discussion

Table 1 summarizes the results of all reported studies (Studies 1 and 2). Among the participants, 12.2, 23.2, and 64.6% of Chinese participants vs. 11.8, 6.6, and 81.6% of American participants chose a declining trend, a stable trend, and an increasing trend, respectively. In general, the majority perceived future climate change to be in an increasing trend. A chi-square test revealed that participants in the two cultures had significantly different patterns in the anticipated climate change, *χ*^2^ (*df* = 2) = 8.71, *p* = 0.013, Cramer’s V = 0.235. Chinese participants were more likely to anticipate a stable trend (Chinese: 23.2% vs. Americans: 6.6%), *p* = 0.004, but less likely to anticipate an increasing trend (Chinese: 64.6% vs. Americans: 81.6%), *p* = 0.017, than were American participants. There was no difference in the frequency of choosing the declining trend between the two cultures (Chinese: 12.2% vs. Americans: 11.8%), *p* = 0.946.

**Table 1:** A summary of the results in the two studies.

	*χ*^2^ (*df* = 2)	Option	Chinese/dialectical condition (% within column)	Americans/analytic condition (% within column)
Study 1	8.71^*^	Declining	12.2_a_	11.8_a_
		Stable	23.2_a_	6.6_b_
		Increasing	64.6_a_	81.6_b_
Study 2	4.99^ɫ^	Declining	43.1_a_	32.8_a_
		Stable	5.2_a_	0_a_
		Increasing	51.7_a_	67.2_a_

Different subscripts within each row indicate a significant difference between the conditions at *p* < 0.05.

ɫ *p* = 0.082;

* *p* < 0.05.

Study 1 provided some evidence indicating that dialectical beliefs affected people’s anticipation of climate change. Despite the fact that the majority of participants perceived an increasing trend for future climate change, Chinese participants, who were from a culture that promotes dialectical thinking, were less likely to anticipate an increasing trend but more likely to anticipate a stable trend for climate change relative to American participants, who were from a culture that promotes linear thinking.

Study 1 had some limitations. First, the sample characteristics were not equivalent between the two cultural groups. For instance, American participants were significantly older than Chinese participants. Second, although abundant evidence supports that Chinese are likely to adopt dialectical thinking, whereas Americans are likely to adopt linear thinking (Ng and Hynie, 2014; Li et al., 2016, 2020; for a review, see Spencer-Rodgers et al., 2010), investigating cultural influences by simply comparing cultural groups was not without limitations, as indicated in previous cross-cultural methodology studies (Fischer and Poortinga, 2018). First of all, there was lack of direct evidence, showing that the difference in perception of future climate change was attributed to dialectical vs. linear thinking, as other well-established cultural factors, such as self-construal (Markus and Kitayama, 1991) and social axioms (Leung and Bond, 2004), could explain the observed cultural differences. In addition, Study 1 did not provide causal evidence showing that dialectical vs. linear thinking leads to the difference in the perception of future climate change.

## Study 2

To address the limitations in Study 1, we manipulated dialectical and linear thinking to test the causal relation between dialectical beliefs and perception of future climate change.

### Participants and Procedure

Following the procedure of estimating the target sample size used in Study 1, we needed at least 108 participants to achieve 80% power for a chi-square test with an expected medium effect size (Faul et al., 2009). Finally, we recruited 118 participants (mean age = 19.54, *SD* = 1.52; 59% female) from a university in China. They were given 10 Chinese Yuan for their participation. The data of two participants were excluded in the final analyses because they did not complete the study, leading to a data set of 116 participants (mean age = 19.53, *SD* = 1.52; 59% female).

**Figure 2:**
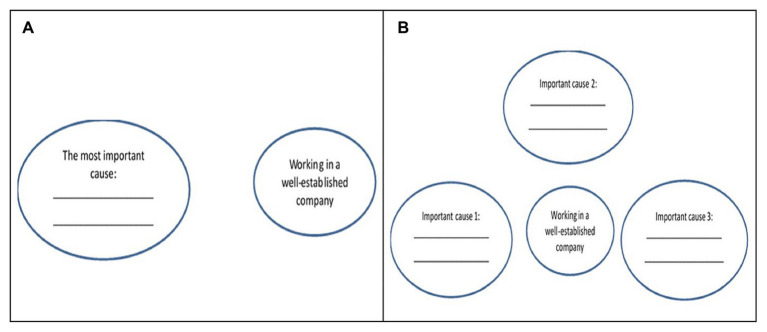
**(A)** The diagram presented in the linear-thinking condition and **(B)** the diagram presented in the dialectical-thinking condition in Study 2.

Participants were randomly assigned to one of the two conditions, dialectical- vs. linear-thinking condition. It took about 10 min to complete the study. A manipulation paradigm was adopted from Spina et al. (2010a, Study 4a) with some modifications. Participants were given the following instruction, “Getting into a competitive company such as Huawei is a major achievement. The majority of fresh graduates do not make it into these famous well-established companies, and a large number of applicants to these big companies such as Huawei are turned away every year.” Next, participants were asked to complete a diagram by providing important cause(s) for this target event (i.e., getting a job in a well-established company; Figure 2). In the linear-thinking condition, participants were asked to provide a major cause that leads to the occurrence of the target event and draw a line to connect the major cause with the event. They were also asked to explain how we could make that important cause available. In the dialectical-thinking condition, participants were asked to provide multiple causes (three causes) that lead to the occurrence of the target event and draw lines to indicate as many or few relationships among the causes and the event as they perceive. They were also asked to explain how these causes can be interconnected with each other.

After completing the manipulation task, the participants were presented with a line chart that shows the real climate change from 1880 to 2010, which was in an increasing trend. Instead of presenting possible trends of climate change like what we did in Study 1, we asked participants to indicate their anticipation of climate change from 2010 to 2060 by continuing the line. The design allows the participants to freely indicate their subjective perception without constraints. We categorized participants’ responses into a declining trend, a stable trend, or an increasing trend for climate change anticipation by comparing the last point provided by us and the last point drawn by the participant.

### Results and Discussion

Similar to Study 1, the majority perceived future climate change to be in an increasing trend, in which 43.1, 5.2, and 51.7% of participants in the dialectical-thinking condition vs. 32.8, 0, and 67.2% of participants in the linear-thinking condition chose a declining trend, a stable trend, and an increasing trend, respectively.

A chi-square test showed that participants in the two conditions had marginally significant differences in the climate change anticipation, *χ*^2^ (*df* = 2) = 4.99, *p* = 0.082, Cramer’s V = 0.207. The results showed that participants in the dialectical-thinking condition were more likely to choose a stable trend (dialectical-thinking condition: 5.2% vs. linear-thinking condition: 0%), *p* = 0.079, but less likely to choose an increasing trend than those in the linear-thinking condition (dialectical-thinking condition: 51.7% vs. linear-thinking condition: 67.2%), *p* = 0.089. However, both results were marginally significant. In contrast, there was no difference in the frequency of choosing a declining trend (dialectical-thinking condition: 43.1% vs. linear-thinking condition: 32.8%), *p* = 0.709.

Study 2 generally replicated the findings obtained in Study 1, showing that participants in the two conditions had marginally significantly different anticipation of climate change. Participants activated with dialectical thinking were more likely to anticipate a stable trend for climate change but less likely to anticipate an increasing trend relative to those activated with linear thinking, whereas there was no significant difference in the likelihood of choosing a declining trend for future climate change between the two conditions.

We estimated the target sample size for Study 2 based on the results of Study 1 (Cramer’s V = 0.235, a medium effect size). Unexpectedly the effect size obtained in this study turned out to be smaller (Cramer’s V = 0.207), which led to marginally significant results. With the minimal manipulation paradigm, i.e., only having a simple task with one round, we observed an effect size that is closer to the medium level in Study 2. We believed that the influence of dialectal beliefs on the perception of climate change should not be neglected, although we need to be cautious that the results were not statistically significant. These marginally significant results for the influence of dialectical beliefs were further discussed in the General Discussion section.

## General Discussion

The current research explored whether dialectical beliefs would shape people’s perception of climate change, an issue that people have acquired prior knowledge globally. Study 1 found significant differences in the anticipation of climate change between Chinese participants, representing people from cultures that promote dialectical thinking, and North American participants, representing people from cultures that promote linear thinking. Specifically, the Chinese participants were less likely to anticipate an increasing trend but more likely to anticipate a stable trend for future climate change relative to the North American participants. To obtain direct causal evidence for the influence of dialectical beliefs on the perception of climate change, Study 2 activated Chinese participants with either dialectical or linear thinking and generally replicated the findings in Study 1, although the results were only marginally significant.

The results of the two studies brought some important messages. First, regardless of the cultural groups or the manipulation conditions, the majority of participants in the two studies anticipated an accelerated climate change in the future. This may implicate the success of climate change education programs globally, in which most people perceive that climate change will get more severe. Second, like for other psychological tendencies or behaviors (e.g., Markus and Kitayama, 1991; Nisbett and Masuda, 2003; Leung and Bond, 2004), culture is found to significantly shape people’s response to different environmental issues (e.g., Milfont et al., 2010; Eom et al., 2016; Pisano and Lubell, 2017; Tam and Chan, 2017). Specifically, the findings in the two studies showed differences in the overall pattern of climate change anticipation. Consistently, people from a culture that promotes dialectical thinking or activated with dialectical thinking were more likely to choose a stable trend but less likely to choose an increasing trend for future climate change as compared with those from a culture that promotes linear thinking or activated with linear thinking.

The current research attempted to overcome some methodological challenges in cross-cultural (environmental) research. First, as Fischer and Poortinga (2018) pointed out, simply comparing responses among multiple cultural groups does not allow us to pinpoint which specific factor(s) may attribute to the observed cultural differences, as cultures can be different in multiple dimensions (e.g., Li et al., 2016). Manipulating a specific cultural mindset, such as independent vs. interdependent self-construal or promotion vs. prevention focus, can address this concern. The current research activated dialectical vs. linear thinking among participants. Replicating other cross-cultural comparison studies (e.g., Spina et al., 2010b; Li et al., 2018), we found some evidence supporting that dialectical vs. linear thinking leads to different responses in climate change anticipation.

Although we found some generally supportive findings in Study 2, the results were only marginally significant due to the unexpected weaker effect of dialectical thinking on the perception of climate change. These findings may be in line with the complex nature of understanding public response to climate change (Weber and Stern, 2011). In fact, multiple factors, ranging from personal factors, such as values, religiosity, confidence in science, and educational attainment (Smith and Leiserowitz, 2013; Lee et al., 2015; Ecklund et al., 2017; Wang and Kim, 2018) to societal factors, such as carbon dioxide emissions (Luis et al., 2018), have been identified to be important determinants of perception of climate change. Thus, it might be likely to observe a weak effect when we consider the influence of one single factor on people’s perception of climate change. To make it more complex, multiple factors that bring the opposite effects may take place when we examine the cultural influences on the perception of climate change. As what we found, linear thinking, which is more prevalent in Western societies (Li et al., 2014, 2020), may make people more likely to anticipate an increasing trend for climate change. In contrast, personal control, which is also more prevalent in Western societies (Ji et al., 2000), may make people less likely to anticipate an increasing trend for climate change, as they may believe that humans’ efforts can substantially change the current threatening trend. To provide a thorough examination of how the identified determinants independently and interactively shape people’s perception of climate change, future studies with considering multiple dimensions simultaneously in one analysis are needed.

The current research may also bring some practical implications for the development of international campaigns for addressing climate change issues. Although the delivery of scientific knowledge of climate change is important for raising public awareness of climate change, Weber and Stern (2011) argued that the public awareness of climate change could be easily affected by people’s habitual thinking. Despite the varied strength of the effect, the present research found that dialectical beliefs tend to make people more likely to perceive an unchanged trend but less likely to perceive an increasing trend for future climate change. Taking together, although the proper knowledge of climate change can be acquired through climate change education, the interpretation of the current increasing trend of global warming can be likely to be biased by the strong influence of culture (i.e., dialectical thinking in the present research) on individuals, as suggested by the present findings. These findings further suggest that the effectiveness of global climate change education programs may be rather limited if we ignore the potential negative influence of cultural factors that substantially shape people’s habitual thinking. Therefore, it is important to incorporate the cross-cultural perspectives into climate change education programs (Perkins et al., 2018). For instance, we may gain insights from cross-cultural research that examines what conditions can weaken the influence of dialectical thinking, which may help to generate possible solutions to remove the influence of dialectical thinking on the perception of climate change. A previous study suggested that the influence of dialectical beliefs on people’s psychological tendency can be eliminated when participants were engaged in an important decision task (vs. in a trivial decision task; Li et al., 2014). Following these findings, we may consider emphasizing the importance of solving environmental issues to remove the influence of dialectical thinking in the international climate change education programs.

### Limitations and Future Directions

There were some limitations in the current research. First, we did not include manipulation-check items in the experiments. Although the paradigm has been proved to be effective in activating dialectical or linear thinking in previous work (Spina et al., 2010a), future studies should include items to ensure the manipulation effect. Second, future studies need to conduct experiments with manipulating dialectical beliefs in more than one culture, which can help to address whether a factor has a universal influence (e.g., Milfont et al., 2010; Hadler and Haller, 2011; Li et al., 2018; Ito and Li, 2019). Third, we used similar measures to assess participants’ anticipation of climate change. Future studies should replicate the results using different measurements to assess people’s perception of future climate change. Additionally, we did not control for the effect of confounds. For instance, the prior knowledge in climate change, which is an important predictor of people’s perception of climate change (Aksit et al., 2018), should be controlled. This concern, however, might be minimal, as previous work found that there were no significant differences in the level of climate change awareness between Westerners and Asians (Muroi and Bertone, 2019). Finally, the current research did not explore whether the difference in perception of future climate change induced by dialectical beliefs would actually lead to some downstream consequences. Given that the perception of climate change was related to pro-environmental tendencies (Panno et al., 2015) and dialectical beliefs were found to be negatively associated with pro-environmental tendencies (Li et al., 2020), it would be possible that perception of future climate change can be a mediator for the relation between dialectical beliefs and pro-environmental tendencies, which can be tested in future studies.

## Conclusion

To summarize, we found consistent findings in the two studies, which revealed different perceptions of future climate change between dialectical thinkers (or people from cultures that promote dialectical thinking) and linear thinkers (or people from cultures that promote linear thinking). Although the majority of participants in both studies anticipated an accelerated climate change, dialectical thinking (vs. linear thinking) made people more likely to anticipate a status quo (a stable trend) but less likely to anticipate an increasing trend for climate change. The current research was the first study to provide causal evidence that demonstrating the influence of dialectical beliefs on anticipation of climate change, which potentially brought some important implications for cross-cultural environmental research and international climate change education programs.

## Data Availability Statement

The raw data supporting the conclusions of this article will be made available by the authors, without undue reservation.

## Ethics Statement

The studies involving human participants were reviewed and approved by Departmental Ethics Committee from the Department of Psychology, Sun Yat Sen University. The patients/participants provided their written informed consent to participate in this study.

## Author Contributions

LL contributed to conceptualization of the study, study design, data analyses, writing and revision, and funding acquisition. DM and W-QL contributed to data collection and revision of the draft. KI contributed to conceptualization of the study, revision of the draft, and funding acquisition. All authors contributed to the article and approved the submitted version.

### Conflict of Interest

The authors declare that the research was conducted in the absence of any commercial or financial relationships that could be construed as a potential conflict of interest.
